# The development of a rapid SYBR Green I-based quantitative PCR for detection of Duck circovirus

**DOI:** 10.1186/1743-422X-8-465

**Published:** 2011-10-07

**Authors:** Chunhe Wan, Yu Huang, Longfei Cheng, Guanghua Fu, Shao-hua Shi, Hongmei Chen, Chunxiang Peng, Fang Lin, Jiansheng Lin

**Affiliations:** 1 Institute of Animal Husbandry and Veterinary Medicine, Fujian Academy of Agricultural Sciences, Fuzhou 350013, China

**Keywords:** duck circovirus, SYBR Green I, real-time PCR

## Abstract

This report describes a one-step real-time polymerase chain reaction assay based on SYBR Green I for detection of a broad range of duck circovirus (DuCV). Align with all DuCV complete genome sequences and other Genus *Circovirus *download from the GenBank (such as goose circovirus, pigeon circovirus), the primers targets to the replicate gene of DuCV were designed. The detection assay was linear in the range of 1.31 × 10^2^-1.31 × 10^7 ^copies/μL. The reaction efficiency of the assay using the slope (the slope was -3.349) and the Y-intercept was 37.01 from the linear equation was estimated to be 0.99 and the correlation coefficient (R^2^) was 0.993. A series of experiments were carried out to assess the reproducibility, sensitivity, and specificity of the assay, following by the low intra-assay and inter-assay CVs for CT values obtained with the standard plasmids. The intra-assay CVs were equal or less than 1.89% and the inter-assay CVs were equal or less than 1.26%. There was no cross-reaction occurred with nucleic acids extracted from RA (Riemerella anatipestifer), E. coli (Escherichia coli), Duck Cholera (Pasteurella multocida), Avian influenza virus, avian paramyxovirus, Muscovy duck parvovirus, Duck reovirus, Duck hepatitis A virus as control templates. The nucleic acids extracted from samples of healthy ducks were used as negative controls. The assay was specific and reproducible. The established real time PCR was used to detect 45 DuCV-negative samples, which were tested using conventional PCR under the developed optimal conditions, each 15 for embryonated eggs, non-embryonated budgerigar eggs, newly hatched duck, the mixture of the lung, liver, spleen which were analysis for the presence of DuCV DNA, to conform that whether the DuCV can be transmitted vertically. Meanwhile, no positive result was shown by the real-time PCR method. The SYBR Green I-based quantitative PCR can therefore be practically used as an alternative diagnostic tool and a screening method for ducks infected with duck circovirus.

## Introduction

Circovirus are small, non-enveloped, icosahedral particles with the diameter of about 20 nm, having a circular single-stranded DNA with approximately 2kilobase in genome size [1]. Currently, the family *Circoviridae *comprised with the two genera *Gyrovirus *and *Circovirus*. The genus *Gyrovirus *contains only the chicken infectious anemia virus (CIAV) [2]. Within the genus *Circovirus *contains several members, including two porcine circovirus types 1 and 2 (PCV1 and PCV2)[3], the psittactine beak and feather disease virus (BFDV)[4], the columbid circorus (CoCV, also known as pigeon circovirus (PiCV)) [5,6], the canary circovirus (CaCV) [7], the goose circovirus (GoCV) [6,8], the duck circovirus (DuCV) [9], the raven circovirus (RaCV) [10], the starling circovirus (StCV) [11], the finch and gull circovirus ((FiCV & GuCV) [12], the ostrich circovirus [13] and recently identified mute swan circovirus (SwCV) which infecting *Cygnus olor *[14].

Duck circovirus (DuCV) had been listed as a tentative member of the Genus circovirus by ICTV. It was reported first in Germany at 2003 [9]. Since then, DuCV was subsequently isolated in Germany [15], Hungary [16], the Taiwan area [17] and the U.S. [18]. We firstly reported the detection of DuCV in Fujian Province, China [19].

Virus isolation is a fundamental diagnostic method, but no in vivo culture system was yet available for propagation of the Genus *Circovirus *except for PCV (type 1 and type 2). Other diagnostic techniques, such as conventional poly-merase chain reaction (PCR) [16], the nested PCR [14] and in situ hybridization (ISH) had been developed. Comparing these techniques, the nested PCR and the ISH were shown to be more sensitive than conventional PCR. However, both assays are labor-intensive; the nested PCR requires agarose gel analysis for the detection of amplification products and had a very high risk of contamination, while the ISH required several days to be done.

Recently, an excellent diagnostic tool with high sensitivity, specificity, and a fast turnaround time had been used extensively for detection of amplicons that are amplified during the PCR cycling in real time. Reports on Genus *Circovirus *virus detection based on real-time PCR technology had been developed [20-22].

In this study, the development of a quantitative real-time PCR for the detection of DuCV based on SYBR Green I dye was reported. To evaluate the developed real-time PCR for diagnosing and monitoring ducks with DuCV infection, we compared the results of conventional PCR and real-time PCR using clinical samples from 29 duck samples distributed in different areas of Fujian Province, China. The clinical symptoms of the sample duck flocks were not obviously abnormal, but sporadic deaths and growth retardation in a small number of ducks were often observed in these sample flocks, also 45 DuCV samples also collected in Fujian Province, each 15 for embryonated eggs, non-embryonated budgerigar eggs, newly hatched duck, the mixture of the lung, liver, spleen which were analyzed for the presence of DuCV DNA, respectively. The establishment of a SYBR Green I-based quantitative PCR can therefore be practically used as an alternative diagnostic tool for duck circovirus infection.

## Materials and methods

### DNA extractions

DNA was isolated from a mixture of the lung, liver, spleen and fabricius bursa of each sample using DNeasy Blood & Tissue kit (Qiagen, Germany) according to the manufacturer's instructions. Total DNA was precipitated and used for DuCV examination, and each DNA sample was defined as a virus isolated.

### Oligonucleotide primers design

Complete genome sequences of DuCV (45 sequences), PiCV (13 sequences) and GoCV (15 sequences) were retrieved from GenBank (see Accession Numbers), and were aligned to identify for the conserved regions using DNAStar software (DNASTAR, Madison, WI). The primers designed for DuCV quantization using SYBR Green I based real-time PCR, based on nucleotide sequences of ORF-V1 (open reading frame, the replicate protein, Rep protein), The primers were as follows: DuCVp1, 5'-TGTTATCTTTGGGCGTGG-3'; DuCVp2, 5'-CATTTCCCGAGTAACCGTC-3'. The length of the amplified products was 191bp [position 459 to 649 at GenBank: EF451157].

### Construction of standard plasmids for real-time PCR

To generate a DuCV standard (pDuCV-ORF-V1) curve for the real-time reaction, a PCR product containing 930 bp [position 4 to 933 at GenBank:EF451157], using the oligo nucleotide primers DuCVp3, 5'-GGCGCTTGTACTCCGTAC-3' and DuCVp4, 5'-TTGGTCTCAGTAGTTTATTGG-3') was cloned into the vector pMD18-T vector (Takara, Dalian, China) according to the instructions of the manufacturer. The resulting plasmid was used to transform *Escherichia coli *DH5α cells and was purified using a QIAGEN plasmid purification kit (Qiagen, Germany) according to the manufacturer's instructions. The concentration of the plasmid preparation was determined by measuring the OD at 260 nm using a spectrophotometer (Eppendorf, Germany). Serial 10-fold dilutions of plasmid DNA with EASY dilution (Takara, Dalian, China) were used in the amplification reactions. The dilutions were stored at -20°C, while stock plasmid was stored at -70°C.

### Real-time PCR

Real-time PCR was done using the Mastercycler^® ^ep realplex system (Eppendorf, Germany) with SYBR Green I detection and *T*m analysis. SYBR^® ^Premix Ex Taq™ (perfect real time) kit was purchased from TaKaRa (Dalian, China). The procedure was optimized with regard to concentrations of primers, and denature/extension temperature. The optimized reaction was carried out in a 20 μL final reaction volume containing10 μL of kit-supplied SYBR^® ^PCR master mix, 0.4 μL concentrations of each forward and reverse primer (each 10 μm), 2 μL DNA solutions, and 7.2 μL distilled water to final volume 20.0 μL. Prior to cycling, the glass capillaries were sealed and centrifuged at 3 000 rpm for 10 s. The thermal profile for the real-time PCR was 95°C for 120 s, followed by 40 cycles of 95°C for 10 s, 60°C for 30 s with two-step. Six concentrations of pDuCV-ORF-V1 (1.31 × 10^2^-1.31 × 10^7 ^DNA copies/μL per sample) were included in each run, and served as positive controls as well as to derive the standard curve used for quantization of DuCV DNA in tissue samples. Positive and negative reference samples were tested along with the unknown samples in each run.

### Melting curve analysis of the PCR product

Melting curve analysis was performed to measure the specificity of PCR product. After PCR cycling, samples were heated to 95°C for 15 s, 65°C for 15 s and then heated to 95°C for15 s at a linear transition rate of 0.1°C/s, and then hold at 16°C. Fluorescence of the samples was monitored continuously while the temperature was increasing. SYBR Green I is released upon denaturation, which results in a decreasing fluorescence of the signal. The software calculates the *T*m. All samples were analyzed once.

### Reproducibility and specificity of the assay

The standard DuCV plasmids (pDuCV-ORF-V1) with (1.31 × 10^2^-1.31 × 10^7 ^DNA copies/μL per sample) were used to evaluate the coefficients of variation (CVs) of the real-time PCR. Intra-assay (three times) and inter-assay (three times for three weeks) CVs for Ct values were both included.

To test the specificity of the assay, nucleic acids extracted from samples of healthy ducks were used as negative controls. The controls also included nucleic acids extracted from RA (Riemerella anatipestifer), *E. coli *(Escherichia coli), duck Cholera (Pasteurella multocida), avian influenza virus, avian paramyxovirus, Muscovy duck parvovirus, duck reovirus, duck hepatitis A virus. No cross-amplification occurred with these controls as templates.

### Conventional PCR reaction

The primer-pair was designed according to Chen [17], with the sequences of forward: 5'-ATATTA TTACCGGCGC(C/T) TGTA-3' and reverse: 5'-TCAGGAATCCCTG (A/C) AGGTGA-3'. The targeted amplification is a 228-bp segment of DuCV genome. Amplifications were programmed as follows by described [17]. Amplicons of 228-bp were separated through 2.0% agarose gel. Positive and negative reference samples were applied in each reaction.

### Detection of clinical samples

9 DuCV-positive samples [GenBank: GQ334371, GQ423740-GQ423747] and 20 DuCV-negative samples were tested using conventional PCR and real-time PCR under optimal conditions. Products from conventional PCR were examined in 2% agarose gel. And also, 45 DuCV-negative samples were tested using conventional PCR under optimal conditions, each 15 for embryonated eggs, non-embryonated budgerigar eggs, newly hatched duck, the mixture of the lung, liver, spleen which were analysis for the presence of DuCV DNA, to conform that whether the DuCV can be transmitted vertically.

## Results

### Real-time PCR for DuCV

**Figure 1:**
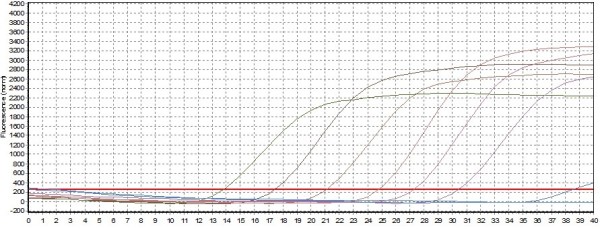
**The specificity and sensitivity of one step SYBR green real-time RT-PCR**. Plot of the amplification of a 10-fold serial dilution (1.31 × 10^7^-1.31 × 10^2^) of pDuCV-ORF-V1 to calculate the detection limit and sensitivity of real-time PCR by analyzing the fluorescence curve of the 191 bp DNA amplification product.

**Figure 2:**
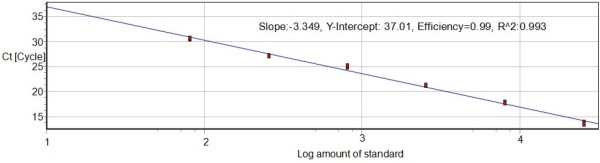
**Real-time PCR standard curve generated from plasmid DNA amplification plot**. Standard curve was plotted in the sample plasmid on the *x*-axis and cycle threshold (Ct) on the *y*-axis. The *x*-axis represents pDuCV-ORF-V1 in 10-fold dilutions and the *y*-axis the fluorescence data used for Ct determinations in dRn (baseline-corrected normalized fluorescence). The assays were linear range of pDuCV-ORF-V1 with *R*2 values (square of the correlation coefficient) of 0.993 and reaction efficiencies of 99%.

Ten-fold serial plasmid dilutions were used to construct the standard curve by plotting the logarithm of the plasmid copy number against the measured Ct values (Figure 1 and Figure 2). The standard curve generated had a wide dynamic range of 1.31 × 10^2^-1.31 × 10^7 ^DNA copies/μL with a linear correlation (*R*2) of 0.993 and efficiency of 0.99 between the Ct value and the logarithm of the plasmid copy number. The reaction efficiency of the assay using the slope (slope = -3.349) and the Y-intercept is 37.01.

### Specificity of the assay

**Figure 3:**
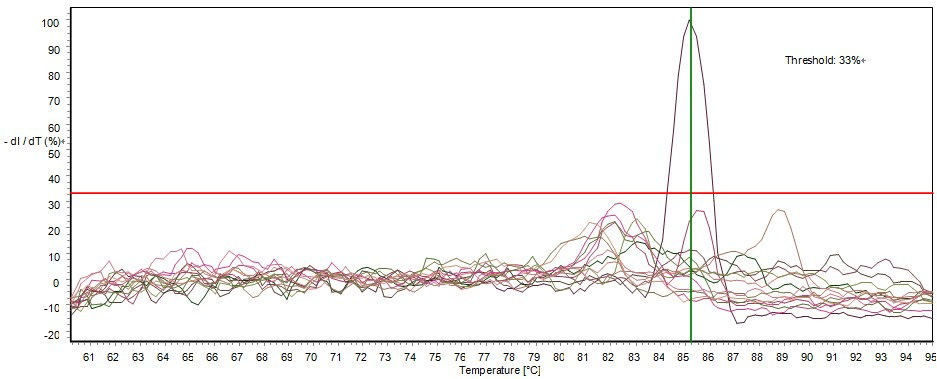
**The specificity of the real-time PCR assay**. Negative control including NTC (no template control), RA, *E. coli*, Duck Cholera, Avian influenza virus (H9 subtype), avian paramyxovirus (AMPV-1), Muscovy duck parvovirus (MDPV), Duck reovirus(DRV), Duck hepatitis A virus (DHV), the positive samples showed an identical melting curve profile. The nucleic acids extracted from samples of healthy ducks were used as negative controls.

The specificity of the assay was examined with regard to the nucleic acids extracted from RA (Riemerella anatipestifer), *E. coli *(Escherichia coli), Duck Cholera (Pasteurella multocida), Avian influenza virus, avian paramyxovirus, Muscovy duck parvovirus, Duck reovirus, Duck hepatitis A virus, were run under the optimal conditions of the assay, and no increase in fluorescence being observed. The nucleic acids extracted from samples of healthy ducks were used as negative controls (Figure 3).

### Reproducibility of the real-time PCR for DuCV

When the standard DuCV plasmid DNA was used for the evaluation of the coefficients of variation (CVs) of the real-time PCR, the intra- and inter-assay CVs for *C*T values ranged between 0.16% and 1.89%, and 0.19% and 1.26%, respectively (Table 1).

**Table 1:** Variance analysis of *C*T values quantified by real-time PCR in serially diluted standard plasmid solutions

Concentration of standard plasmid (DNA copies/μL)	Intra-assay variability			Inter-assay variability		
	*C*T		CV (%)	*C*T		CV (%)
	Mean	SD		Mean	SD	
1.31 × 10^7^	13.67	0.26	1.89	13.59	0.17	1.26
1.31 × 10^6^	17.66	0.19	1.07	17.62	0.13	0.74
1.31 × 10^5^	21.19	0.03	0.16	21.20	0.04	0.19
1.31 × 10^4^	25.00	0.13	0.50	25.06	0.08	0.32
1.31 × 10^3^	27.22	0.17	0.62	27.23	0.20	0.72
1.31 × 10^2^	30.61	0.18	0.60	30.41	0.34	1.10

The coefficients of variation (CVs) of the real-time PCR, the intra-assay (three times) and inter-assay (three times for three weeks) CVs for Ct values were both included, the intra- and inter-assay CVs for *C*T values ranged between 0.16% - 1.89%, and 0.19% - 1.26%, respectively.

### Comparison the results obtained by real-time PCR and conventional PCR

Results of the real-time PCR and conventional PCR assays are shown in Table 2. All positive samples for DuCV DNA in conventional PCR were also positive in real-time PCR assay, whereas 7 in 20 of the samples negative for DuCV DNA in the conventional PCR assays were positive in the real-time PCR assays.

**Table 2:** Comparison of real-time PCR and conventional PCR assay results

Conventional PCR assay results		real-time PCR assay results		
		Positive	Negative	Total
Positive		9	0	9
Negative	20	7	13	20
	45^#^	0	45	45
Total		16	58	74

^# ^These 45 samples described at Materials and methods, detection of clinical samples for conform that whether the DuCV can be transmitted vertically. And also no positive was shown by the optimized real-time PCR.

## Discussion

Avian circovirus, a good indicator of immunosuppressive threats to avian species, infections were originally diagnose depending on histology or electron microscopy, which requiring specialist skills or equipment. Histological diagnosis of circoviruses usually involves the detection of characteristic botryoid basophilic inclusions that commonly occur within the cytoplasm of macrophages present in lymphoid tissue such as fabricius bursa, but some published reports showed it had some obvious associated clinical characteristics, e.g. a feathering disorder, poor body condition and low weight, which may induce damage to lymphoid tissue and immunosuppression [9,15]. Therefore, other diagnostic techniques, such as in situ hybridization (ISH), conventional poly-merase chain reaction (PCR), and nested PCR were important tools for avian circovirus epidemiological investigation.

A SYBR Green I-based quantitative PCR is an excellent diagnostic tool with high sensitivity, specificity, and a fast turnaround time [23,24]. This system is called real-time PCR because the accumulated amplicons can be monitored directly during the DNA amplification process in closed tube with no post-PCR electrophoresis by a real-time PCR method. In addition, the real-time PCR technique has been shown to provide good sensitivity and a linear relationship between the copy number and cycle threshold (Ct) values. The quantization of DNA is based on the determination of the threshold cycle when the amplified PCR product is first detected. The higher the initial DNA copy number input, the sooner the product of amplification is detected.

The real-time PCR increased the detection of DuCV samples over that achieved by conventional PCR (table 2). Tests on the reproducibility and specificity of the method suggest that the established real-time PCR system appears to be reliable and stable. A series of experiments were carried out to assess the reproducibility, sensitivity, and specificity of the assay, following by the low intra-assay and inter-assay CVs for *C*T values obtained with the standard plasmids. The intra-assay CVs were equal or less than 1.89% and the inter-assay CVs were equal or less than 1.26%. No cross-reaction signals were detected when using several common duck diseases as the negative controls, which demonstrated not only the specificity of the assay but also the specific for the primer-set. DuCV infection and co- infection was common in duck farms and had been reported in DuCV-infected ducks without any clinical symptoms [25,26]. The ability to quantify viral loads with the quantitative PCR assay was valuable tools to gain further understands of mechanisms of DuCV infection. All 45 samples described at Materials and methods, Detection of clinical samples for conform that whether the DuCV can be transmitted vertically, however, no positive result was shown by the conventional PCR and real-time PCR. Though, the BFDV DNAs were detected can be transmitted horizontally and vertically [27].

The primers used in this study were positioned in ORF-V1 (Rep gene) that was conserved when in parewising comparisons of 45 different DuCV complete genomic sequences, which belonged to two genotypes. While, the first real-time PCR detected for DuCV was described by Fringuelli E et al [16], with only one DuCV full-genome sequence [9,15], though also selected from the circovirus Rep gene. And also, there are some avian viruses had establishment using a TaqMan-based real-time PCR [20,28-30]. With these primer sets, our PCR assay can detect all DuCV identified by the real-time PCR. Moreover, SYBR Green I can bind to any double-strand DNA, so the dye can also be used in diagnosis of other viruses, and most of real-time machines can detect the fluorescence emitted by SYBR Green I. These will lower the diagnosis costs and make the method more applicable and practicable than probe. The real-time PCR detection system complements and extends previous methods for detection and quantization of duck circovirus.

The SYBR Green I-based quantitative PCR can also be applied to evaluate for the ducks infected with duck circovirus.

## Accession Numbers

The DuCV accession numbers used in this manuscript for oligonucleotide primers design: AY228555, AY394721, DQ100076, DQ166836-DQ166838, EF370476, EF451157, GU168779, EU022374-EU022375, EU344802-EU344807, EU499309-EU499311, FJ554673, GQ334371, GQ423740-GQ423747, GQ868757, GU014543, GU131340-GU131343, HM162345-HM162353.

The PiCV accession numbers used in this manuscript for oligonucleotide primers design: AF252610, AJ298229-AJ298230, EU840176, DQ090944-DQ090945, DQ915956-DQ915961, JN183455.

The GoCV accession numbers used in this manuscript for oligonucleotide primers design: AF418552, AF536931-AF536936, AJ304456, AY633653, DQ192283-DQ192287, GU320569.

## Competing interests

The authors declare that they have no competing interests.

## Authors' contributions

CW and YH designed and performed the majority of the experiments in this study. CW wrote the paper. LC, GF, SS, HC, CP, FL and JL had made substantial contributions to detection of the clinical samples. All authors read and approved the final manuscript.

## References

[B1] Todd D Avian circovirus diseases: lessons for the study of PMWS Vet Microbiol 2004 98 169 174 10.1016/j.vetmic.2003.10.010 14741130

[B2] Hailemariam H Omar A Hair-Bejo M Giap T Detection and characterization of chicken anemia virus from commercial broiler breeder chickens Virol J 2008 5 128 10.1186/1743-422X-5-128 18954433 PMC2605446

[B3] Faurez F Dory D Grasland B Jestin A Replication of porcine circoviruses Virol J 2009 18 60 10.1186/1743-422X-6-60 PMC2690592 19450240

[B4] Raidal SR Riddoch PA A feather disease in Senegal doves (Streptopelia senegalensis) morphologically similar to psittacine beak and feather disease Avian Pathol 1997 26 829 836 10.1080/03079459708419256 18483948

[B5] Mankertz A Hattermann K Ehlers B Soike D Cloning and sequencing of columbid circovirus (coCV), a new circovirus from pigeons Arch Virol 2000 145 2469 2479 10.1007/s007050070002 11205099

[B6] Todd D Weston JH Soike D Smyth JA Genome sequence determinations and analyses of novel circoviruses from goose and pigeon Virology 2001 286 354 362 10.1006/viro.2001.0985 11485403

[B7] Pheix KV Weston JH Ypelaar I Lavazza A Smyth JA Todd D Wilcox GE Ridal SR Nucleotide sequence analysis of a novel circvirus of canaries and its relationship to other members of the genus Circovirus of the family Circoviridae J Gen Virol 2001 82 2805 2809 11602793 10.1099/0022-1317-82-11-2805

[B8] Soike D Kohler B Albrecht K A circovirus-like infection in geese related to a runting syndrome Avian Pathol 1999 28 199 202 10.1080/03079459994939 26911506

[B9] Hattermann K Schmitt C Soike D Mankertz A Cloning and sequencing of Duck circovirus (DuCV) Arch Virol 2003 148 2471 2480 10.1007/s00705-003-0181-y 14648300

[B10] Stewart ME Perry R Raidal SR Identification of a novel circovirus in Australian ravens (Corvus coronoides) with feather disease Avian Pathol 2006 35 86 92 10.1080/03079450600597345 16595298

[B11] Johne R Fernández-de-Luco D Höfle U Müller H Genome of a novel circovirus of starlings, amplified by multiply primed rolling-circle amplification J Gen Virol 2006 87 1189 1195 10.1099/vir.0.81561-0 16603520

[B12] Todd D Scott AN Fringuelli E Shivraprasad HL Gavier-Widen D Smyth JA Molecular characterization of novel circoviruses from finch and gull Avian Pathol 2007 36 75 81 10.1080/03079450601113654 17364513

[B13] Eisenberg SW van Asten AJ van Ederen AM Dorrestein GM Detection of circovirus with a polymerase chain reaction in the ostrich (Struthio camelus) on a farm in The Netherlands Vet Microbiol 2003 95 27 38 10.1016/S0378-1135(03)00122-6 12860074

[B14] Halami MY Nieper H Müller H Johne R Detection of a novel circovirus in mute swans (Cygnus olor) by using nested broad-spectrum PCR Virus Res 2008 132 208 212 10.1016/j.virusres.2007.11.001 18082907

[B15] Soike D Albrecht K Hattermann K Schmitt C Mankertz A Novel circovirus in mulard ducks with developmental and feathering disorders Vet Rec 2004 154 792 793 10.1136/vr.154.25.792 15233459

[B16] Fringuelli E Scott AN Beckett A McKillen J Smyth JA Palya V Glavits R Ivanics E Mankertz A Franciosini MP Todd D Diagnosis of duck circovirus infections by conventional and real-time polymerase chain reaction tests Avian Pathol 2005 34 495 500 10.1080/03079450500368334 16537165

[B17] Chen C Wang P Lee M Shien J Shien H Ou S Chen C Chang P Development of a polymerase chain reaction procedure for detection and differentiation of duck and goose circovirus Avian Dis 2006 50 92 95 10.1637/7435-090705R1.1 16617989

[B18] Banda A Galloway-Haskins RI Sandhu TS Schat KA Genetic analysis of a duck circovirus detected in commercial Pekin ducks in New York Avian Dis 2007 51 90 95 10.1637/0005-2086(2007)051[0090:GAOADC]2.0.CO;2 17461272

[B19] Fu G Cheng L Shi S Peng C Chen H Huang Y Genome Cloning and Sequence Analysis of Duck Circovirus Chin J Virol 2008 24 138 143 [in Chinese] 18533346

[B20] Zhao K Han F Zou Y Zhu L Li C Xu Y Zhang C Tan F Wang J Tao S He X Zhou Z Tang X Rapid detection of porcine circovirus type 2 using a TaqMan-based real-time PCR Virol J 2010 7 374 10.1186/1743-422X-7-374 21192832 PMC3023794

[B21] Shearer PL Sharp M Bonne N Clark P Raidal SR A quantitative, real-time polymerase chain reaction assay for beak and feather disease virus J Virol Methods 2009 159 98 104 10.1016/j.jviromet.2009.03.009 19442852

[B22] Duchatel JP Todd D Willeman C Losson B Quantification of pigeon circovirus in serum, blood, semen and different tissues of naturally infected pigeons using a real-time polymerase chain reaction Avian Pathol 2009 38 143 148 10.1080/03079450902737805 19322713

[B23] Yang Z Habib M Shuai J Fang W Detection of PCV2 DNA by SYBR Green I-based quantitative PCR J Zhejiang Univ Sci B 2007 8 162 169 10.1631/jzus.2007.B0162 17323427 PMC1810386

[B24] Tian H Wu J Shang Y Cheng Y Liu X The development of a rapid SYBR one step real-time RT-PCR for detection of porcine reproductive and respiratory syndrome virus Virol J 2010 7 90 10.1186/1743-422X-7-90 20459705 PMC2874540

[B25] Zhang X Jiang S Wu J Zhao Q Sun Y Kong Y Li X Yao M Chai T An investigation of duck circovirus and co-infection in Cherry Valley ducks in Shandong Province, China Vet Microbiol 2009 133 252 256 10.1016/j.vetmic.2008.07.005 18760549

[B26] Wang D Xie X Zhang D Ma G Wang X Zhang D Detection of duck circovirus in China: A proposal on genotype classification Vet Microbiol 2011 147 410 415 10.1016/j.vetmic.2010.07.014 20709471

[B27] Rahaus M Dwsloges N Probst S Loebbert B Lantermann W Wolff MH Detection of beak and feather disease virus DNA in embryonated eggs of psittacine birds Vet Med-Czech 2008 53 53 58

[B28] Guo Y Cheng A Wang M Shen C Jia R Chen S Zhang N Development of TaqMan^® ^MGB fluorescent real-time PCR assay for the detection of anatid herpesvirus 1 Virol J 2009 6 71 10.1186/1743-422X-6-71 19497115 PMC2696427

[B29] Zou Q Sun K Cheng A Wang M Xu C Zhu D Jia R Luo Q Zhou Y Chen Z Chen X Detection of anatid herpesvirus 1 gC gene by TaqMan fluorescent quantitative real-time PCR with specific primers and probe Virol J 2010 7 37 10.1186/1743-422X-7-37 20152046 PMC2837632

[B30] Song C Zhu C Zhang C Cui S Detection of porcine parvovirus using a taqman-based real-time pcr with primers and probe designed for the NS1 gene Virol J 2010 7 353 10.1186/1743-422X-7-353 21126330 PMC3014914

